# Supplementary material for: UBR-1 enzyme network regulates glutamate homeostasis to affect organismal behavior and developmental viability
Source: bioRxiv. 2025 Jul 30:2025.07.28.666006. Preprint. [Version 1] doi: 10.1101/2025.07.28.666006 (PMC12324394; doi:10.1101/2025.07.28.666006)
Supplement: Supplement 7 [file NIHPP2025.07.28.666006v1-supplement-7.pdf]

bioRxiv preprint doi: https://doi.org/10.1101/2025.07.28.666006; this version posted July 30, 2025. The copyright holder for this preprint (which was not certified by peer review) is the author/funder, who has granted bioRxiv a license to display the preprint in perpetuity. It is made available under aCC-BY 4.0 International license.

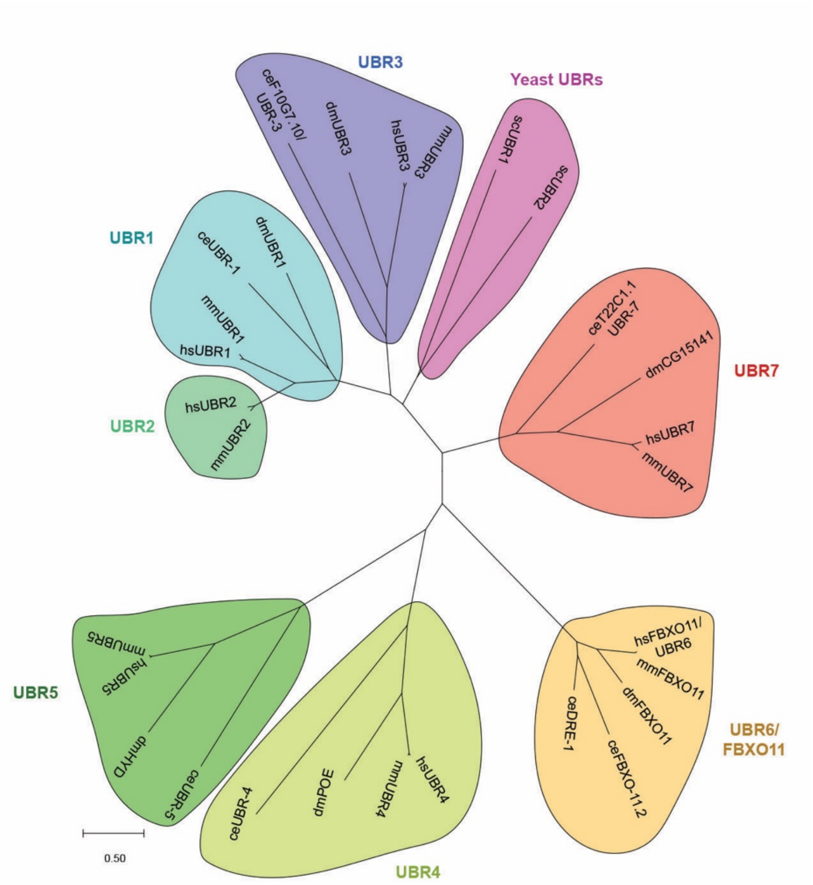

Supplementary Fig 1: **UBR family proteins are conserved across phylogenetic clades from yeast through mammals.** Phylogenetic tree shows UBR protein family, including *C. elegans* UBR-1 and its mammalian orthologs UBR1 and UBR2, are conserved from single-celled eukaryotes through vertebrates. Protein sequences from yeast (*Saccharomyces cerevisiae*, sc), *C. elegans* (ce), fruitfly (*Drosophila melanogaster*, dm), mouse (*Mus musculus*, mm), and human (*Homo sapiens*, hs) were aligned using MUSCLE and built into a maximum likelihood phylogenetic tree using MEGA11 maximum likelihood analysis method.

bioRxiv preprint doi: https://doi.org/10.1101/2025.07.28.666006; this version posted July 30, 2025. The copyright holder for this preprint (which was not certified by peer review) is the author/funder, who has granted bioRxiv a license to display the preprint in perpetuity. It is made available under aCC-BY 4.0 International license.

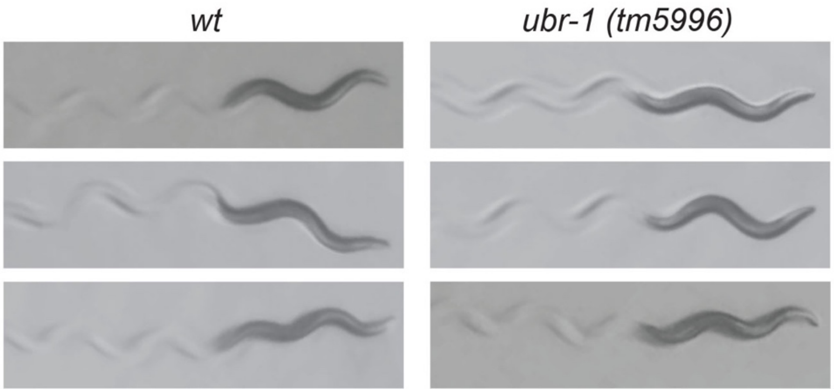

Supplementary Fig 2: *ubr-1* mutants do not display forward locomotion defects during low-intensity locomotion on solid media. Still frame images showing *C. elegans* and tracks on solid media plates for indicated genotypes. Note *ubr-1(tm5996)* mutants display similar forward locomotion to wild type on solid media. Animals were placed on a solid-media growth plate and allowed to crawl for 1 min before recording.

bioRxiv preprint doi: https://doi.org/10.1101/2025.07.28.666006; this version posted July 30, 2025. The copyright holder for this preprint (which was not certified by peer review) is the author/funder, who has granted bioRxiv a license to display the preprint in perpetuity. It is made available under aCC-BY 4.0 International license.

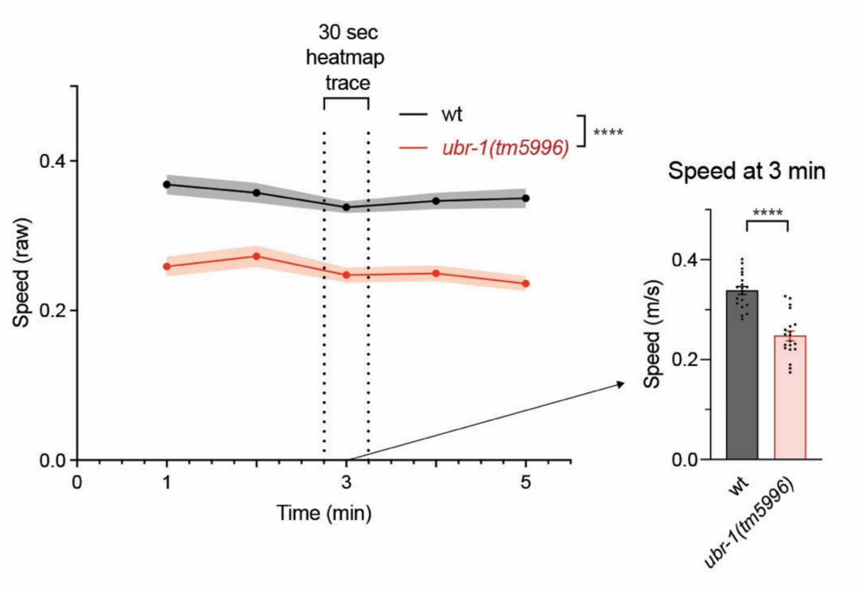

Supplementary Fig 3: Expanded analysis of UBR-1 effects on high-intensity locomotor speed at early time points. Quantitation of high-intensity swimming locomotor speeds for wild type and *ubr-1* mutants across time window used to create MWT traces of individual animals shown in **Figure 1D**. 30 sec Heatmap trace time window for **Figure 1D** is annotated (between dashed lines). Shown are MWT plots of average locomotor speed (left) and expanded quantitation at 3 minutes (right). MWT plots (solid lines, left) represent average speed of all recorded animals (4 animals/well, 5 wells per genotype per experiment, and 3-5 independent experiments) and shaded regions are SEM. Bars represent average of all wells for indicated time point, dots represent single wells tracked (4 animals/well), and error bars are SEM. Significance for plots (genotype annotations, left) tested with pairwise two-way ANOVA, and significance for bars with dots (right) tested using an unpaired two-tailed Student's *t*-test. **** p<0.0001

bioRxiv preprint doi: https://doi.org/10.1101/2025.07.28.666006; this version posted July 30, 2025. The copyright holder for this preprint (which was not certified by peer review) is the author/funder, who has granted bioRxiv a license to display the preprint in perpetuity. It is made available under aCC-BY 4.0 International license.

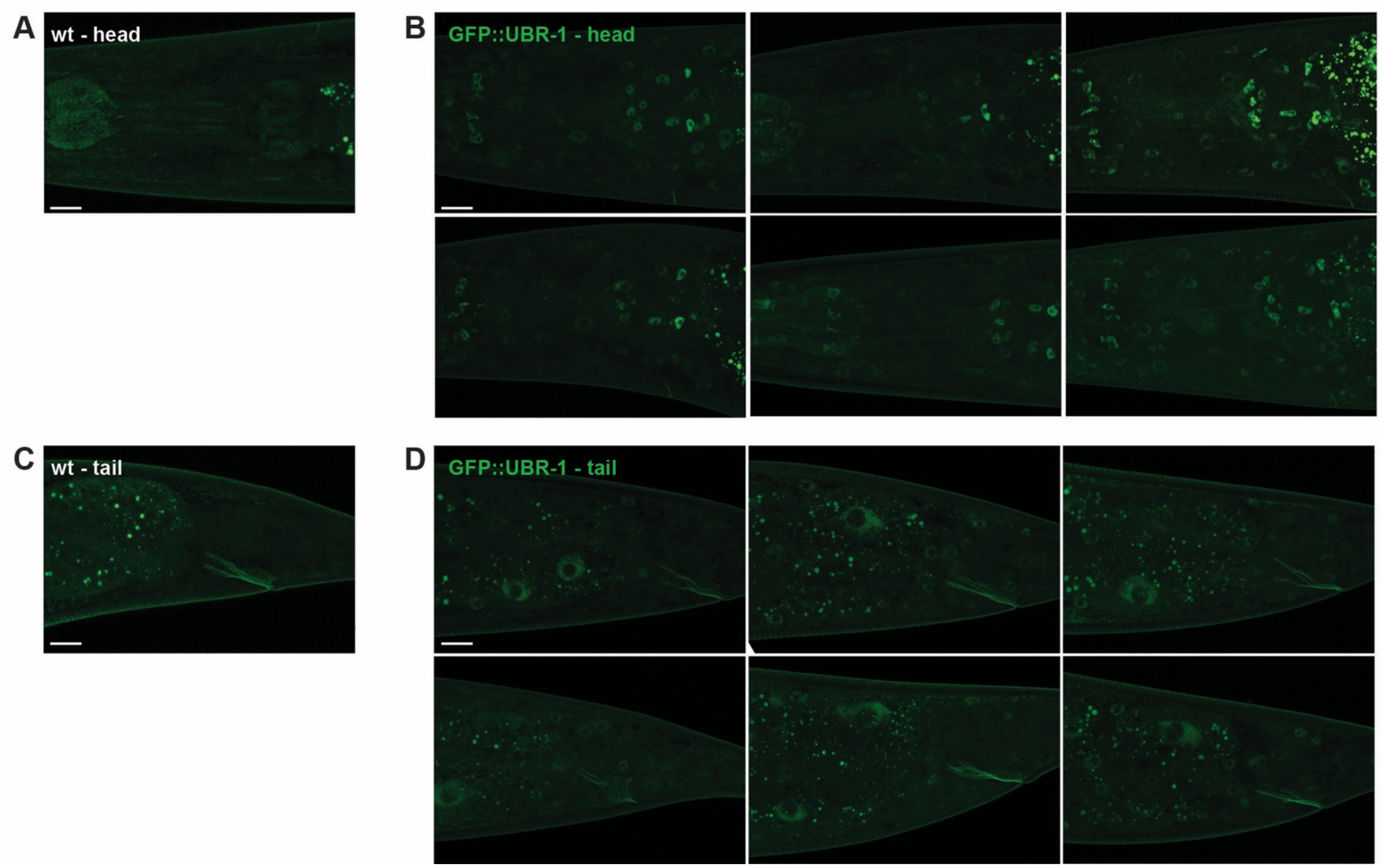

Supplementary Fig 4: **Expanded dataset showing CRISPR engineered GFP::UBR-1 expression in head and tail neurons. A**. Super-resolution image of adult *C. elegans* head. In wild type, autofluorescence shows animal's body outline, pharynx, and gut granules (bright puncta far right). **B**. Further examples of super-resolution images showing GFP::UBR-1 expression in head neurons, continued from **Figure 2C**. Note GFP::UBR-1 is excluded for neuronal nuclei. **C**. Super-resolution image of tail in wild type where autofluorescence shows animal's body outline, anus and gut granules (bright puncta left). **D**. Examples of Super-resolution images showing GFP::UBR-1 expression in tail neurons.

Scale bar is 10 μm

bioRxiv preprint doi: https://doi.org/10.1101/2025.07.28.666006; this version posted July 30, 2025. The copyright holder for this preprint (which was not certified by peer review) is the author/funder, who has granted bioRxiv a license to display the preprint in perpetuity. It is made available under aCC-BY 4.0 International license.

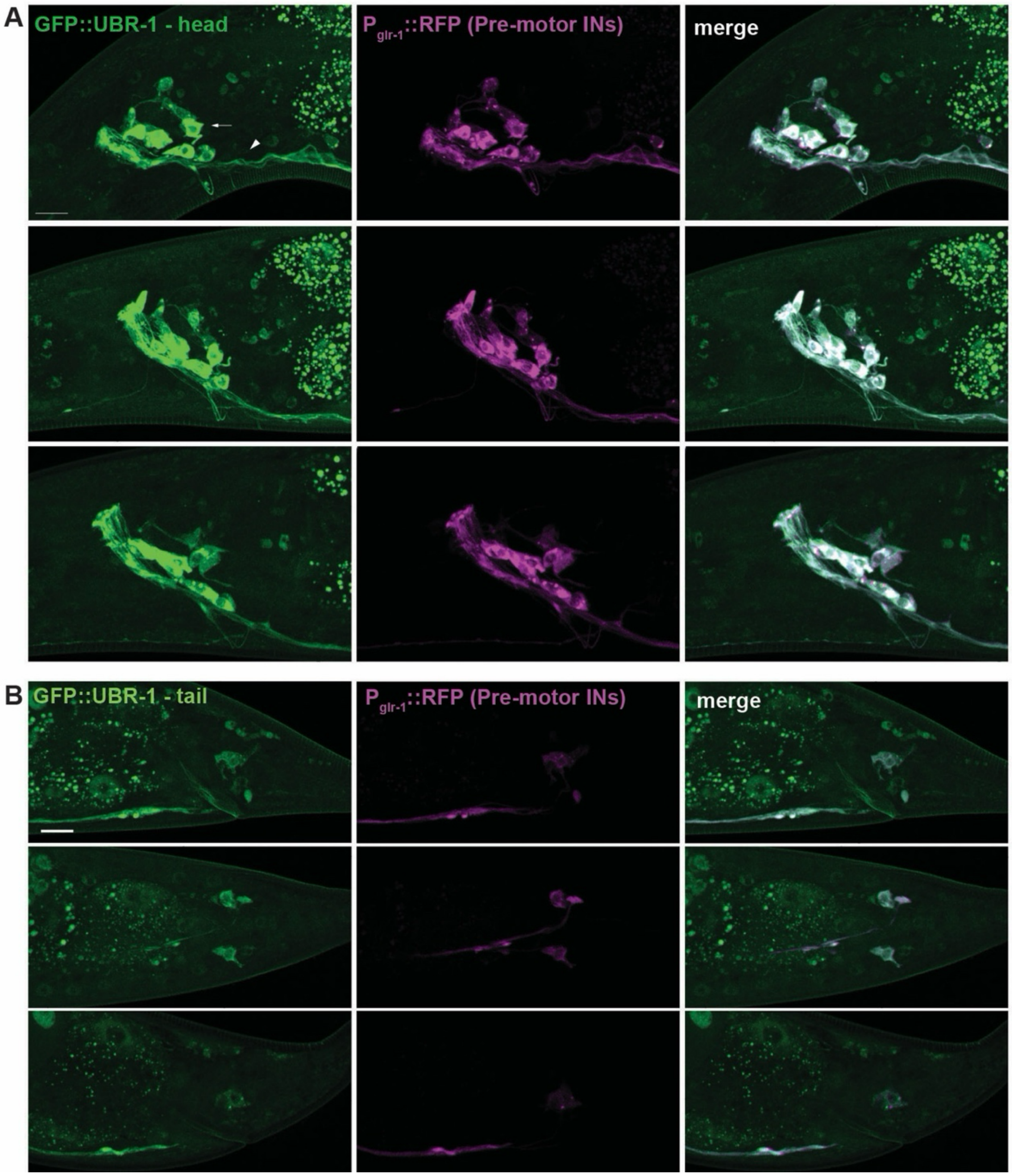

Supplementary Fig 5: **Expanded dataset showing CRISPR engineered GFP::UBR-1 expression in pre-motor interneurons. A and B**. Super-resolution images of GFP::UBR-1 (green) localized to cell bodies (arrows) and axons (arrowhead) of pre-motor interneurons in head (**A**) and tail (**B**). Note GFP::UBR-1 is excluded for neuronal nuclei. Interneurons visualized using P*glr-1*::RFP (magenta). Scale bar is 10 μm.

bioRxiv preprint doi: https://doi.org/10.1101/2025.07.28.666006; this version posted July 30, 2025. The copyright holder for this preprint (which was not certified by peer review) is the author/funder, who has granted bioRxiv a license to display the preprint in perpetuity. It is made available under aCC-BY 4.0 International license.

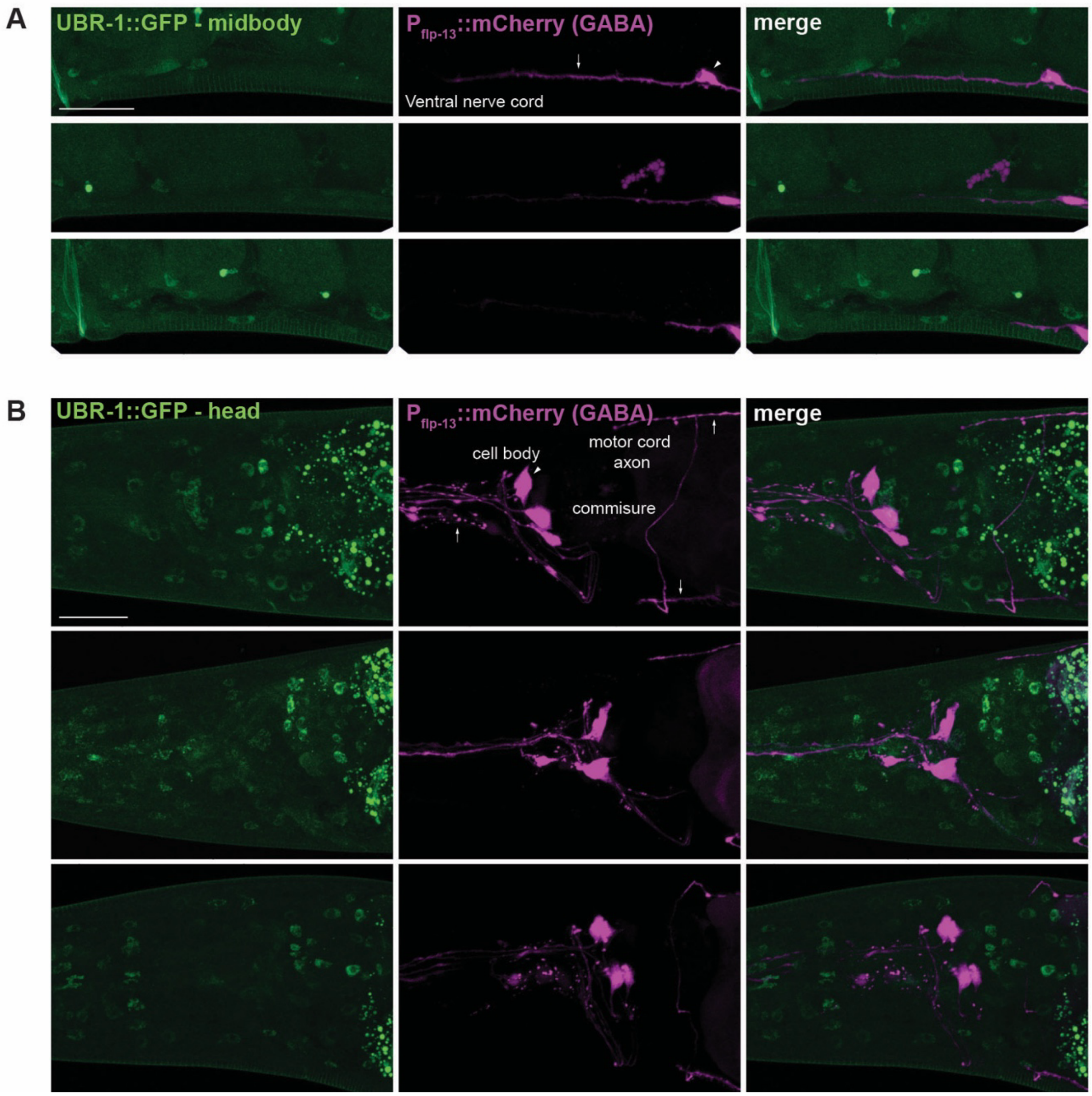

Supplementary Fig 6: **CRISPR engineered GFP::UBR-1 is not expressed in GABA motor neurons. A-B**. Super-resolution images showing GFP::UBR-1 (green) does not localize to cell bodies (arrows) or axons (arrowheads) of inhibitory GABA DD motor neurons in ventral cord (**A**) or GABA neurons in head (**B**). GABA neurons visualized using $P_{flp\text{-}13}$::mCherry (magenta). Scale bar is 10 µm.

bioRxiv preprint doi: https://doi.org/10.1101/2025.07.28.666006; this version posted July 30, 2025. The copyright holder for this preprint (which was not certified by peer review) is the author/funder, who has granted bioRxiv a license to display the preprint in perpetuity. It is made available under aCC-BY 4.0 International license.

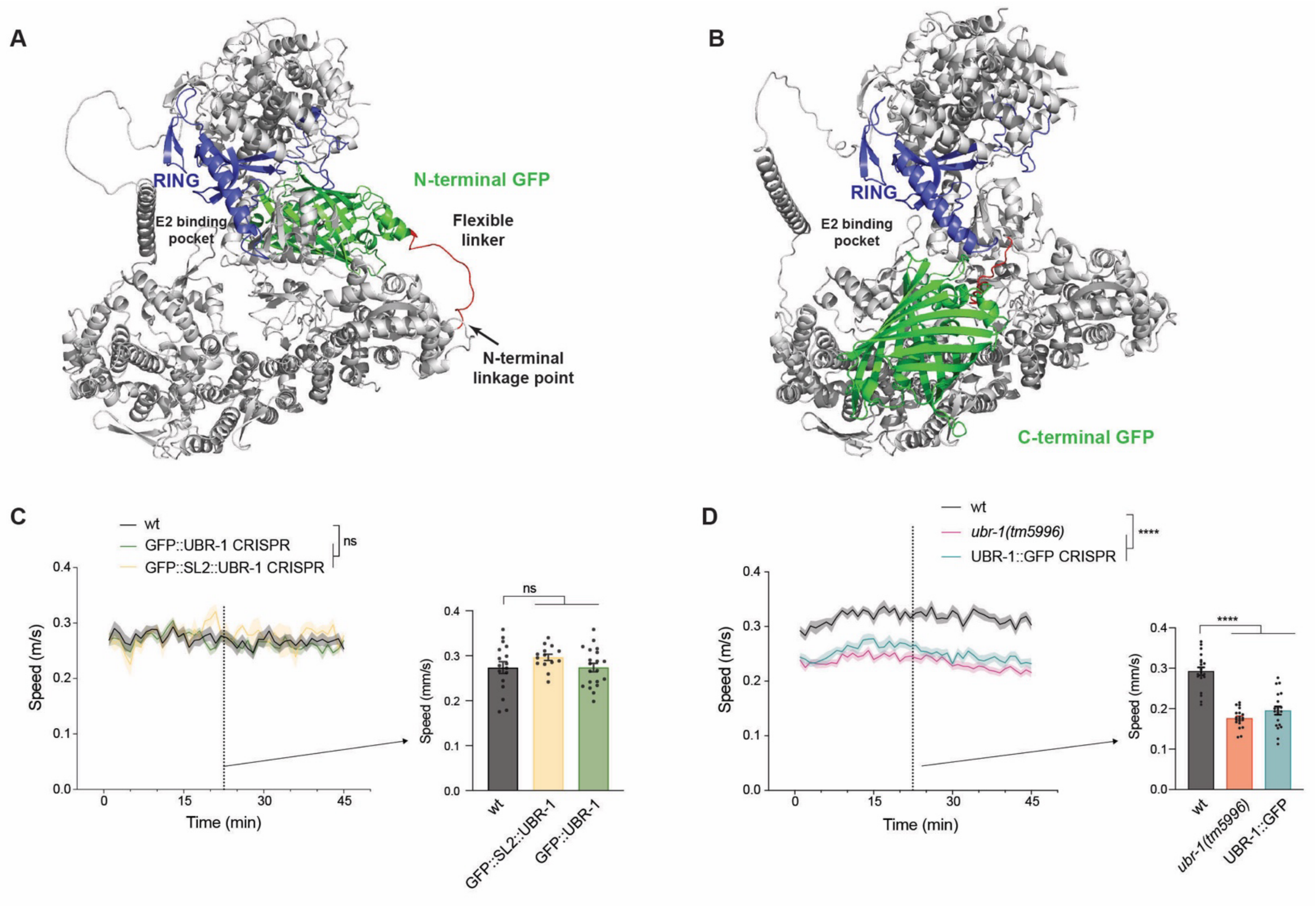

Supplementary Fig 7: Evaluating CRISPR engineered GFP tag location on UBR-1 function. **A and B**: AlphaFold predicted structures of N-terminal-tagged GFP::UBR-1 (**A**) and C-terminal-tagged UBR-1::GFP (**B**). Highlighted are GFP (green), catalytic RING domain (blue) and flexible linker (red). N-terminal GFP is away from RING domain. C-terminal GFP is positioned near catalytic RING suggesting it could sterically hinder binding of E2 or ubiquitin that is transferred to substrate. Note disordered regions were removed from structural prediction. **C**. Quantitation shows locomotor speed is not significantly altered in GFP::UBR-1 CRISPR engineered animals compared to GFP::SL2::UBR-1 negative control or wild type. Shown are MWT plots of average locomotor speed (left) and expanded quantitation at 23 minutes (right). **D**. Quantitation shows UBR-1::GFP CRISPR engineered animals have impaired locomotor speed similar to *ubr-1*

bioRxiv preprint doi: https://doi.org/10.1101/2025.07.28.666006; this version posted July 30, 2025. The copyright holder for this preprint (which was not certified by peer review) is the author/funder, who has granted bioRxiv a license to display the preprint in perpetuity. It is made available under aCC-BY 4.0 International license.

mutants. **For C and D**, MWT plots (solid lines, left) represent average speed of all recorded animals (4 animals/well, 5 wells per genotype per experiment, and 3-5 independent experiments) and shaded regions are SEM. Bars represent average of all wells for indicated time point, dots represent single wells tracked (4 animals/well), and error bars are SEM. Significance for plots (genotype annotations, left) tested with pairwise two-way ANOVA, and significance for bars with dots (right) tested using one-way ANOVA and Bonferroni's post-hoc correction. **** $p<0.0001$, ns = not significant

bioRxiv preprint doi: https://doi.org/10.1101/2025.07.28.666006; this version posted July 30, 2025. The copyright holder for this preprint (which was not certified by peer review) is the author/funder, who has granted bioRxiv a license to display the preprint in perpetuity. It is made available under aCC-BY 4.0 International license.

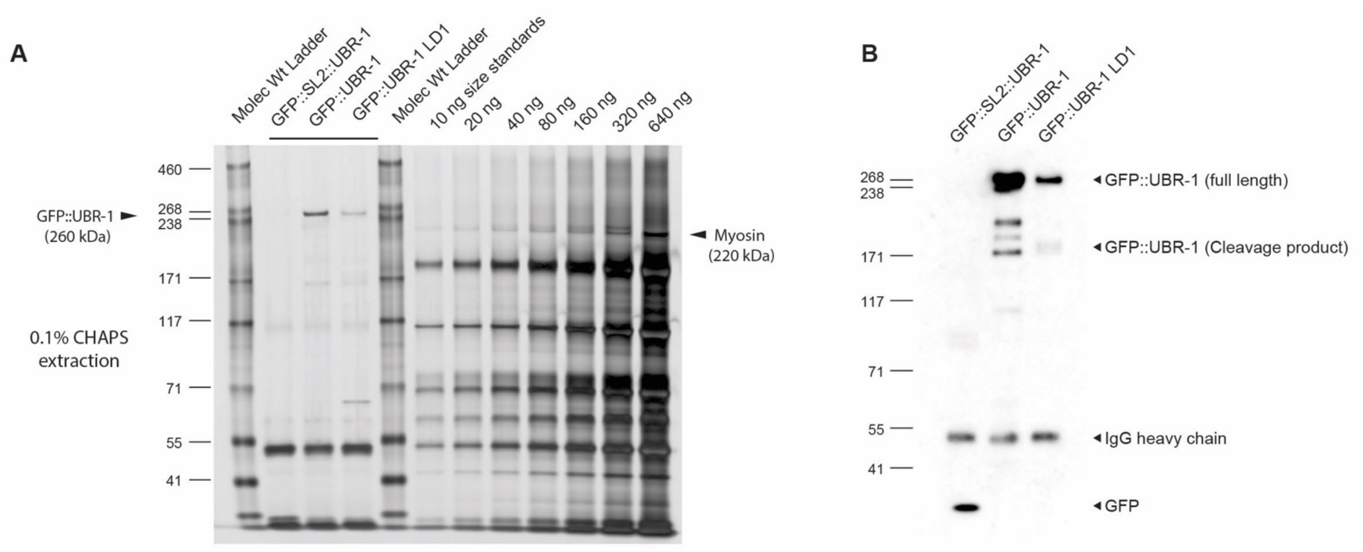

Supplementary Fig 8: Expanded dataset for quality control of *C. elegans* proteomics.

A and B. Further examples of silver stain (**A**) and immunoblot (**B**) of anti-GFP precipitated samples used to evaluate sample quality prior to proteomics for GFP::UBR-1 and GFP::SL2::UBR-1 (negative control). Titrated Myosin standards were used to quantify amount of GFP::UBR-1 to ensure sufficient purification target was present for proteomics. Note GFP::UBR-1 LD1 construct was also evaluated in this preparation.

bioRxiv preprint doi: https://doi.org/10.1101/2025.07.28.666006; this version posted July 30, 2025. The copyright holder for this preprint (which was not certified by peer review) is the author/funder, who has granted bioRxiv a license to display the preprint in perpetuity. It is made available under aCC-BY 4.0 International license.

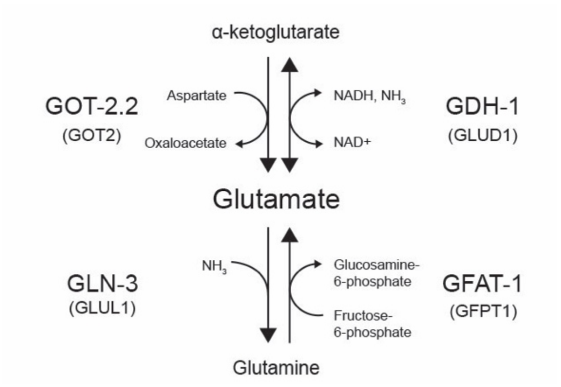

Supplementary Fig 9: Overview of GOT-2.2, GLN-3, GFAT-1 and GDH-1 effects on glutamate metabolism in *C. elegans*. Schematic of glutamate metabolism showing GOT-2.2 and GFAT-1 promote generation of glutamate. GLN-3 has an opposing role converting glutamate to glutamine. GDH-1 catalyzes reversible reaction of glutamate to α-ketoglutarate.

bioRxiv preprint doi: https://doi.org/10.1101/2025.07.28.666006; this version posted July 30, 2025. The copyright holder for this preprint (which was not certified by peer review) is the author/funder, who has granted bioRxiv a license to display the preprint in perpetuity. It is made available under aCC-BY 4.0 International license.

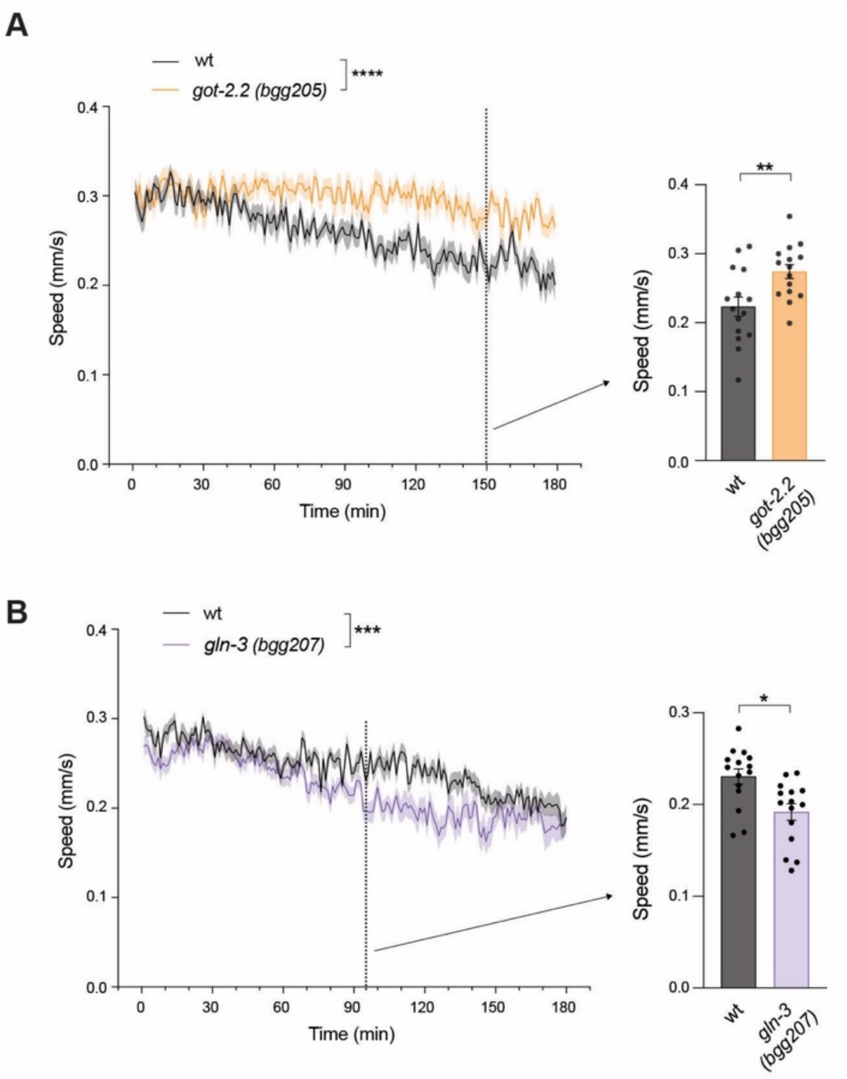

Supplementary Fig 10: Expanded dataset showing further mutant alleles of *got-2* and *gln-3* affect high-intensity locomotor activity. **A**. Quantitation of swimming speed shows sustained locomotion in second mutant allele of *got-2.2 (bgg205)* compared to wild type. Shown are MWT plots of average locomotor speed (left) and expanded quantitation at 150 mins (right). **B**. Quantitation shows second mutant allele of *gln-3 (bgg207)* displays early fatigue compared to wild type. Shown are MWT plots of average locomotor speed (left) and expanded quantitation at 95 mins (right). **For A and B**, MWT plots (solid lines, left) represent average speed of all recorded animals (4 animals/well, 5 wells per genotype per experiment, and 3 independent experiments)

bioRxiv preprint doi: https://doi.org/10.1101/2025.07.28.666006; this version posted July 30, 2025. The copyright holder for this preprint (which was not certified by peer review) is the author/funder, who has granted bioRxiv a license to display the preprint in perpetuity. It is made available under aCC-BY 4.0 International license.

and shaded regions are SEM. Bars represent average of all wells for indicated time point, dots represent single wells tracked (4 animals/well), and error bars are SEM. Significance for plots (genotype annotations, left) tested with pairwise two-way ANOVA, and significance for bars with dots (right) tested using unpaired two-tailed Student's *t*-test. **** $p<0.0001$, *** $p<0.001$, ** $p<0.01$, * $p<0.05$

bioRxiv preprint doi: https://doi.org/10.1101/2025.07.28.666006; this version posted July 30, 2025. The copyright holder for this preprint (which was not certified by peer review) is the author/funder, who has granted bioRxiv a license to display the preprint in perpetuity. It is made available under aCC-BY 4.0 International license.

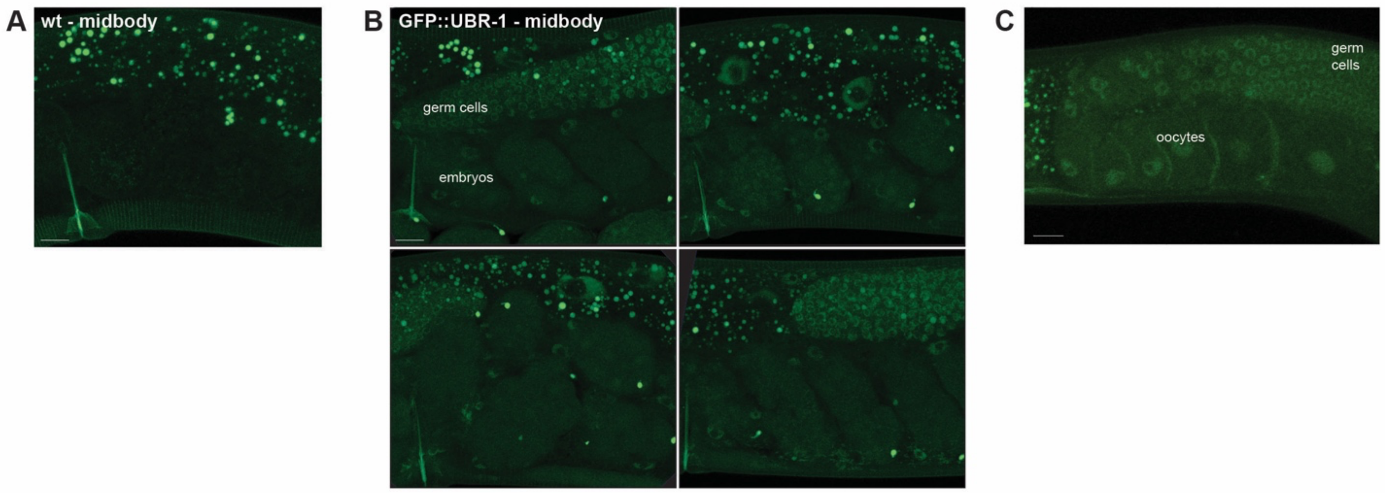

Supplementary Fig 11: Expanded dataset showing CRISPR engineered GFP::UBR-1 expression in gonad. A. Super-resolution image of adult *C. elegans* midbody. In wild type, autofluorescence shows body outline, vulva and gut granules (bright puncta far right). **B**. Further examples of super-resolution images showing GFP::UBR-1 expression in germ cells and embryos. **C**. Further examples of GFP::UBR-1 expression in oocytes and germ cells. **For B and C**, images are continued examples from **Figure 6B and C.** Scale bar is 10 μm.

bioRxiv preprint doi: https://doi.org/10.1101/2025.07.28.666006; this version posted July 30, 2025. The copyright holder for this preprint (which was not certified by peer review) is the author/funder, who has granted bioRxiv a license to display the preprint in perpetuity. It is made available under aCC-BY 4.0 International license.

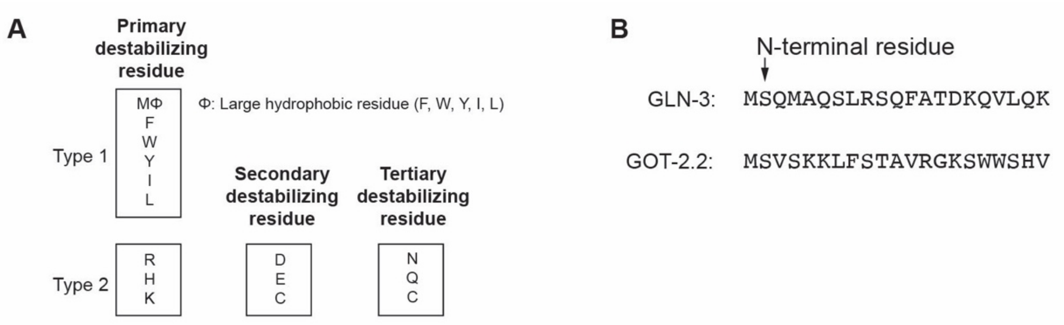

Supplementary Fig 12: GLN-3 and GOT-2.2 do not have predicted UBR-1 substrate recognition degron sequences. A. Diagram of known primary, secondary, and tertiary N-terminal destabilizing residues (N-degrons) recognized by UBR-box proteins to trigger N-end rule ubiquitination and degradation. **B**. N-terminal amino acid sequence for GLN-3 and GOT-2.2. Note first residues following initial methionine are not classified as N-degrons for both proteins.

bioRxiv preprint doi: https://doi.org/10.1101/2025.07.28.666006; this version posted July 30, 2025. The copyright holder for this preprint (which was not certified by peer review) is the author/funder, who has granted bioRxiv a license to display the preprint in perpetuity. It is made available under aCC-BY 4.0 International license.

Supplementary Table 1: CeNGEN transcriptional profiles for *ubr-1* and glutamate metabolic enzymes. CeNGEN single cell neural transcriptional atlas for *C. elegans* was analyzed and expression of *ubr-1*, *gdh-1*, *got-2.2*, *gln-3*, and *gfat-1* were found in pre-motor interneurons, other interneurons and sensory neurons.

Neuron Type	Neuron name	Gene name/expression level (A.U.)				
		ubr-1	*gdh-1*	*got-2.2*	*gln-3*	*gfat-1*
Premotor interneurons	AVA	28.9453953	88.87205	103.337278	0	2.68073386
	AVB	12.9410329	59.4744179	33.8643077	0	3.7898285
	AVD	17.2035832	139.198217	130.954644	0	5.00744669
	AVE	26.6016773	49.2110534	40.6149692	0	1.03052153
	PVC	28.8142887	111.638966	38.8126997	0	2.64676443
Other Interneurons	DVC	45.1575655	140.255778	72.8624164	611.27402	0
	SAA	163.589955	73.3049585	30.4343688	86.8016088	0
	AVG	20.7438498	150.440802	58.7259884	62.0294768	5.9251839
Mechanosensory	ALM	25.3143465	84.1813342	80.2138355	0	0
	AVM	76.3313818	16.8437954	36.948134	0	6.32296964
	PLM	19.5968782	43.424666	50.163687	0	2.18564101
	PVM	13.6372259	76.9372187	40.6103998	0	0
Chemosensory	ADF	15.1839678	51.8934088	8.50809338	274.686346	0
	AQR	9.60767271	33.4824012	47.957631	7.23124595	0
	ASEL	13.5861823	75.1013465	37.798161	17.4823466	0
	ASER	8.8902393	25.8167853	66.9347955	10.0400571	0
	ASK	6.7839966	14.8159511	12.9450041	17.5739975	1.38074102
	AWB	16.1632449	37.6855511	29.1298913	59.2365625	0
	AWC_OFF	3.93569086	28.2716865	36.1801682	18.6682729	0
	PHB	25.0232669	25.8734345	19.0916224	46.7409514	0

bioRxiv preprint doi: https://doi.org/10.1101/2025.07.28.666006; this version posted July 30, 2025. The copyright holder for this preprint (which was not certified by peer review) is the author/funder, who has granted bioRxiv a license to display the preprint in perpetuity. It is made available under aCC-BY 4.0 International license.

Supplementary Table 2: Expanded dataset for UBR-1 proteomic hits.

Mass spectrum data are presented for each individual experiment where glutamate metabolic enzymes were identified as UBR-1 proteomic hits. Shown is percent coverage of protein sequences for individual hits in GFP::UBR-1 test samples. Normalized spectral index (SI_N) for each test sample was calculated using StPeter as a label-free quantitative measure of relative abundance for protein hits where smaller values represent higher abundance.

Identified Protein	Vertebrate Homolog	Molec Wt (kDa)	Detergent Extraction Condition and Experiment	SL2 Spectra (control)	GFP::UBR-1 Total Spectra (test)	# Unique Peptides (test)	% Protein Coverage (test)	Test Normalized Spectral Index (SI_N)
GDH-1	GLUD1	58.8	0.1% NP-40 Experiment 1		26	22	43.8	-16.55
			0.1% NP-40 Experiment 2		8	7	13.8	-17.25
			0.1% CHAPS Experiment 2		6	6	12.9	-18.70
			0.1% Triton Experiment 2	3	12	11	23.7	-17.18
			0.1% NP-40 Experiment 3	17	91	32	72.2	-17.5208
			0.1% NP-40 Experiment 4	15	75	29	60.6	-16.8976
GOT-2.2	GOT2	45.6	0.1% NP-40 Experiment 1		10	9	22.5	-17.807974
			0.1% NP-40 Experiment 2		5	5	11.4	-18.139897
			0.1% CHAPS Experiment 2		3	3	7.5	-18.847915
			0.1% NP-40 Experiment 3	5	39	17	64.3	-18.2337
			0.1% NP-40 Experiment 4	4	21	7	24.2	-17.9864
GLN-3	GLUL	43.6	0.1% NP-40 Experiment 1		8	8	24.7	-18.166422
			0.1% NP-40 Experiment 2		2	2	8	-18.673544
			0.1% NP-40 Experiment 3	6	31	18	63.9	-19.2082
			0.1% NP-40 Experiment 4		21	13	38.9	-18.5937
GFAT-1	GFPT1	81.1	0.1% NP-40 Experiment 1		3	3	4.5	-21.040674

bioRxiv preprint doi: https://doi.org/10.1101/2025.07.28.666006; this version posted July 30, 2025. The copyright holder for this preprint (which was not certified by peer review) is the author/funder, who has granted bioRxiv a license to display the preprint in perpetuity. It is made available under aCC-BY 4.0 International license.

			0.1% CHAPS Experiment 2		6	5	7.7	-18.358251
			0.1% NP-40 Experiment 3		16	5	23.0	-20.9063
			0.1% NP-40 Experiment 4		6	4	8.6	-21.8557

Supplementary Table 3: Transgenic and CRISPR Strains

Figure	Strain	Genotype
Figs 2, 3, 6, Supp Figs 7, 8, 11	XMN1324	*ubr-1*(*bgg110*[GFP::UBR-1 CRISPR]) I
Supp Figs 7, 8	XMN1326	*ubr-1*(*bgg112*[UBR-1::GFP CRISPR]) I
Fig 3, Supp Fig 7	XMN1388	*ubr-1*(*bgg140*[GFP::SL2::UBR-1 CRISPR]) I
Fig 1, Supp Fig 8	XMN1565	*ubr-1*(*bgg212*[LD1 CRISPR CRISPR]) I
Fig 1	XMN1566	*ubr-1*(*bgg116*[LD2 CRISPR CRISPR]) I
Figs 4, 6	XMN1567	*got-2.2*(*bgg204*[3-frame stop CRISPR]) X
Fig 5 Supp Fig 10	XMN1568	*got-2.2*(*bgg205*[3-frame stop CRISPR]) X
Figs 4, 5, 7	XMN1569	*gln-3*(*bgg206*[3-frame stop CRISPR]) IV
Supp Fig 10	XMN1570	*gln-3*(*bgg207*[3-frame stop CRISPRi]) IV
Fig 4, 5	XMN1571	*ubr-1*(*tm5996*) I; *got-2.2*(*bgg208*[3-frame stop CRISPR]) X
Figs 4, 5, 7	XMN1572	*ubr-1*(*tm5996*) I; *gln-3*(*bgg210*[3-frame stop CRISPR]) IV
Fig 1	XMN1573	*ubr-1*(*tm5996*) I; bggSi56[P_{ubr-1}::UBR-1::ubr-1 3'UTR, unc-119(+)] II
Fig 2	XMN1574	*ubr-1*(*tm5996*) I; bggSi57[P_{glr-1}::UBR-1::let-858 3'UTR, unc-119(+)] II
Fig 2, Supp Fig 5	XMN1576	*ubr-1*(*bgg110*[GFP::UBR-1 CRISPR]) I; odIs6(P_{glr-1}::mRFP)
Supp Fig 6	XMN1577	*ubr-1*(*bgg110*[GFP::UBR-1 CRISPR]) I; bggIs6(P_{flp-13}::mCherry)

Supplementary Table 4 – Primers

Gene	Allele/ Construct	Primer Sequence
**Genotyping:**		
ubr-1	*tm5996*	*tm5996* fwd: 5' ACGTTCGGAACACAAAGAGC 3'<br>*tm5996* rev: 5' ACAGCACTTCGACCTTCTCG3'
ubr-1	*bgg110*	common fwd: 5' AGCTCTTTATCTGCGGCAAC 3'<br>*bgg110* rev: 5' TCGAGAAGCATTGAACACCA 3'<br>wt rev: 5' GACACATGTCCCATCAGTGG 3'
ubr-1	*bgg112*	Common fwd: 5' TTGATGACGACACGAAAACG 3'<br>*bgg112* rev: 5' TCGAGAAGCATTGAACACCA3'<br>wt rev: 5' ACTGAATCCCCAACAGTTCC3'
ubr-1	*bgg140*	*bgg140* fwd: 5' ACGACTCACTAGTGGGCAGATCTCAACAAAATACTCCAATTGGC3'<br>*bgg140* rev: 5' GACACATGTCCCATCAGTGG3'
ubr-1	*bgg116, bgg212*	*bgg116/212* fwd: 5' AAAACAAGCCAGCACAATCC 3'<br>*bgg116/212* rev: 5' ACCCATTTTGATTCGTCAGC 3'<br>*bgg116*: digest with AatII<br>*bgg212*: digest with NdeI
ubr-1	*bggSi56*	Genotyping for presence of Mos1 element:<br>left homology: 5' GCGAAAATGAACAGGAAAAAG 3'

bioRxiv preprint doi: https://doi.org/10.1101/2025.07.28.666006; this version posted July 30, 2025. The copyright holder for this preprint (which was not certified by peer review) is the author/funder, who has granted bioRxiv a license to display the preprint in perpetuity. It is made available under aCC-BY 4.0 International license.

		right homology: 5' CGTTATTTTGGAGAAAAACTCG 3'<br>integrated unc-119 rescue rev: 5' AGGAACAGAATAACAGATGATGAGC 3'<br>non-integrated Mos1 element rev: 5' GCTCAATTCGCGCCAAACTAT 3'<br><br>Genotyping for correct integrated transgene:<br>left integration site fwd: 5' GATATTCGAAAAGAGGCAGAATGTG 3'<br>left integration site rev: 5' AGGAACAGAATAACAGATGATGAGC 3'<br>right integration site fwd: 5' AGAAAAATCTTCGAAACGACG 3'<br>right integrate site rev: 5' CACGCCTTCCCTTGTTCAGTCAC 3'
ubr-1	*bggSi57*	Genotyping for presence of Mos1 element:<br>left homology: 5' GCGAAAATGAACAGGAAAAAG 3'<br>right homology: 5' CGTTATTTTGGAGAAAAACTCG 3'<br>integrated unc-119 rescue rev: 5' AGGAACAGAATAACAGATGATGAGC 3'<br>non-integrated Mos1 element rev: 5' GCTCAATTCGCGCCAAACTAT 3'<br><br>Genotyping for correct integrated transgene:<br>left integration site fwd: 5' GATATTCGAAAAGAGGCAGAATGTG 3'<br>left integration site rev: 5' AGGAACAGAATAACAGATGATGAGC 3'<br>right integration site fwd: 5' CGTATTCCGCTCCTCTCATC 3'<br>right integration site rev: 5' CACGCCTTCCCTTGTTCAGTCAC 3'
got-2.2	*bgg204, bgg205, bgg208*	*bgg204/205/208* fwd: 5' CTGGGATAAGATGAGCAGAC3'<br>*bgg204/205/208* rev: 5' GGAAGTCCTGCAAACAAATAA
gln-3	*bgg206, bgg207, bgg210*	*bgg206/207/210* fwd: 5' GACATTGACGGACATCCATT3'<br>*bgg206/207/210* rev: 5' ACTGATCTCCCATCTGGATT3'
**Cloning:**		
ubr-1	pBG-GY1131	attB1r: 5' GGGGACAACTTTGTATAGAAAAGTTGTTGATGGA GTTGCTGACACTG 3'<br>attB4: 5' GGGGACTGCTTTTTTGTACAAACTTGTTCCATTAG TCGAACTGAAATCG 3'
glr-1	pBG-GY1136	attB1r: 5' GGGGACAGCTTTCTTGTACAAAGTGGTTAATTTGTTACTCG CTGAAAACATTATATGC 3'<br>attB4: 5' GGGGACAACTTTGTATAATAAAGTTGATCCCTCTTTGAG TTTCATTCGAAC 3'
ubr-1	pBG-GY1133	attB1: 5' GGGGACAAGTTTGTACAAAAAAGCAGGCTTTATGATTGTCG ATTTAATACAATCAGC 3'<br>attB2: 5' GGGGACCACTTTGTACAAGAAAGCTGGGTTCTAGAAATGAC CCCAATCGTATTG 3'
ubr-1	pBG-GY1134	attB2r: 5' GGGGACAGCTTTCTTGTACAAAGTGGTTAGAAAAATCT TCGAAACGACG 3'<br>5' attB3: GGGGACAACTTTGTATAATAAAGTTGTCCTTAAATTAAT CAGACATTTTATTCAGTGC 3'
let-858	pBG-GY827	attB2r: 5' GGGGACAGCTTTCTTGTACAAAGTGGGGATGATCGACGCC AACGTCGTTG 3'<br>attB3: 5' GGGGACAACTTTGTATAATAAAGTTGCCAAGCGAGGACAAT TCTCATCG 3'
**CRISPR:**		
ubr-1	*bgg110*	Repair fwd: 5' CACTGAAGAAGTTGTGAACTACAATGAAATTGACTTCAG AAATGTGCAATACGATTGGGGTCATTTCGGAGGAGGAGGATCTGGTGGT 3'<br>Repair rev: 5' CAATTTTAATTGGCACCGTCGTTTCGAAGATTTTTCTCTA TTTGTATAGTTCGTCCATGCC 3'
ubr-1	*bgg112*	Repair fwd: 5' TTTTATCAAGCTACCGATTTCAGTTCGACTAATGGAATG AGTAAAGGAGAAGAACTTTTCACTGG 3'<br>Repair rev: 5' GCTTGAGCAGCAGCTGGCGTACTTGGGCCCATTCACCCT GTCGTGCACTTTGTATTAAATCGACAATCATTGATCCACCTCCACCAGA TCCTCCACCACC 3'

bioRxiv preprint doi: https://doi.org/10.1101/2025.07.28.666006; this version posted July 30, 2025. The copyright holder for this preprint (which was not certified by peer review) is the author/funder, who has granted bioRxiv a license to display the preprint in perpetuity. It is made available under aCC-BY 4.0 International license.

Supplementary Table 5 – Plasmids

Plasmid name	Allele	Vector	Description
pBG-GY827	*bggSi57* [P_{glr-1}::UBR-1::let-858 3'UTR, unc-119(+)] II	pDONR P2R-P3	BP clonase II reaction of *let-858* 3'UTR PCR product with pDONR P2R-P3 (see primers)
pBG-GY1131	*bggSi56* [P_{ubr-1}::UBR-1::ubr-1 3'UTR, unc-119(+)] II	pDONR P4-P1R	BP clonase II reaction of P_{ubr-1} PCR product with pDONR P4-P1R (see primers)
pBG-GY1133	*bggSi56/bggSi57*	pDONR221	BP clonase II reaction of genomic *ubr-1* PCR product with pDONR221 (see primers)
pBG-GY1134	*bggSi56*	pDONR P2R-P3	BP clonase II reaction of *ubr-1* 3'UTR PCR product with pDONR P2R-P3 (see primers)
pBG-GY1136	*bggSi57*	pDONR P4-P1R	BP clonase II reaction of P_{glr-1} PCR product with pDONR P4-P1R (see primers)
pBG-GY1139	*bggSi56*	pCFJ150(MosSci (ttTI5605) unc-119 GY(f) R4-R3)	LR recombination of pBG-GY1131, pBG-GY1133, and pBG-GY113 with pCFJ150
pBG-GY1143	*bggSi57*	pCFJ150(MosSci (ttTI5605) unc-119 GY(f) R4-R3)	LR recombination of pBG-GY1136, pBG-GY1133, and pBG-GY827 with pCFJ150

Supplementary Table 6 – CRISPR reagents

Gene	Construct	crRNA Target Sequence + PAM	Repair Template
ubr-1	GFP::UBR-1	TTAATACAATCAGCTCGACA **GGG**	TTTTATCAAGCTACCGATTTCAGTTCGACTAATGGAATGAGTAAAGGAGAAGAACTTTTCACTGGAGTTGTCCCAATTCTTGTTGAATTAGATGGTGATGTTAATGGGCACAAATTTTCTGTCAGTGGAGAGGGTGAAGGTGATGCAACATACGGAAAACTTACCCTTAAATTTATTTGCACTACTGGAAAACTACCTGTTCCATGGGTAAGTTTAAACATATATATACTAACTAACCCTGATTATTTAAATTTTCAGCCAACACTTGTCACTACTTTCTGTTATGGTGTTCAATGCTTCTCGAGATACCCAGATCATATGAAACGGCATGACTTTTTCAAGAGTGCCATGCCCGAAGGTTATGTACAGGAAAGAACTATATTTTTCAAAGATGACGGGAACTACAAGACACGTAAGTTTAAACAGTTCGGTACTAACTAACCATACATATTTAAATTTTCAGGTGCTGAAGTCAAGTTTGAAGGTGATACCCTTGTTAATAGAATCGAGTTAAAAGGTATTGATTTTAAAGAAGATGGAAACATTCTTGGACACAAATTGGAATACAACTATAACTCACACAATGTATACATCATGGCAGACAAACAAAAGAATGGAATCAAAGTTGTAAGTTTAAACATGATTTTACTAACTAACTAATCTGATTTAAATTTTCAGAACTTCAAAATTAGACACAACATTGAAGATGGAAGCGTTCAACTAGCAGACCATTATCAACAAAATACTCCAATTGGCGATGGCCCTGTCCTTTTACCAGACAACCATTACCTGTCCACACAATCTGCCCTTTCGAAAGATCCCAACGAAAAGAGAGACCACATGGTCCTTCTTGAGTTTGTAACAGCTGCTGGGATTACACATGGCATGGACGAACTATACAAAGGAGGAGGAGGATCTGGTGGTGGAGGATCTGGTGGAGGTGGATCAATGATTGTCGATTTAATACAAAGTGCACGACA**GGG**TGAATGGGCCCAAGTACGCCAGCTGCTGCTCAAGC (double-stranded template generated by PCR)

bioRxiv preprint doi: https://doi.org/10.1101/2025.07.28.666006; this version posted July 30, 2025. The copyright holder for this preprint (which was not certified by peer review) is the author/funder, who has granted bioRxiv a license to display the preprint in perpetuity. It is made available under aCC-BY 4.0 International license.

ubr-1	UBR-1::GFP	CCCAATCGTATTGCACATTG **CGG** (reverse strand)	CACTGAAGAAGTTGTGAACTACAATGAAATTGACTT**C**A**G**AAATGTGCAATACGATTGGGGTCATTTCGGAGGAGGAGGATCTGGTGGTGGAGGATCTGGTGGAGGTGGATCAATGAGTAAAGGAGAAGAACTTTTCACTGGAGTTGTCCCAATTCTTGTTGAATTAGATGGTGATGTTAATGGGCACAAATTTTCTGTCAGTGGAGAGGGTGAAGGTGATGCAACATACGGAAAACTTACCCTTAAATTTATTTGCACTACTGGAAAACTACCTGTTCCATGGGTAAGTTTAAACATATATATACTAACTAACCCTGATTATTTAAATTTTCAGCCAACACTTGTCACTACTTTCTGTTATGGTGTTCAATGCTTCTCGAGATACCCAGATCATATGAAACGGCATGACTTTTTCAAGAGTGCCATGCCCGAAGGTTATGTACAGGAAAGAACTATATTTTTCAAAGATGACGGGAACTACAAGACACGTAAGTTTAAACAGTTCGGTACTAACTAACCATACATATTTAAATTTTCAGGTGCTGAAGTCAAGTTTGAAGGTGATACCCTTGTTAATAGAATCGAGTTAAAAGGTATTGATTTTAAAGAAGATGGAAACATTCTTGGACACAAATTGGAATACAACTATAACTCACACAATGTATACATCATGGCAGACAAACAAAAGAATGGAATCAAAGTTGTAAGTTTAAACATGATTTTACTAACTAACTAATCTGATTTAAATTTTCAGAACTTCAAAATTAGACACAACATTGAAGATGGAAGCGTTCAACTAGCAGACCATTATCAACAAAATACTCCAATTGGCGATGGCCCTGTCCTTTTACCAGACAACCATTACCTGTCCACACAATCTGCCCTTTCGAAAGATCCCAACGAAAAGAGAGACCACATGGTCCTTCTTGAGTTTGTAACAGCTGCTGGGATTACACATGGCATGGACGAACTATACAAATAGAGAAAAATCTTCGAAACGACGGTGCCAATTAAAATTG (double-stranded template generated by PCR)
ubr-1	GFP::SL2::UBR-1	TCTGGTGGTGGAGGATCTGG **TGG**	CTATACAAAGGAGGAGGAGGATCTGGTGGTGGAGGTAGTGGA**GG**AGGTGGATCATAATGGTCTCTTCTCAATAAAGGTTGTATATTTATTCATCTTATTGAATCTGCCATTTCCTCGTTTTTGCGAGTTTATATACCTTCCAATTTTCTTTCTATTGTATTTTCAACTTCTAATTTTAATTCAGGGAAACTGCTTCAACGCATCATGATTGTCGATTTAATACAAAGTGCACGACAGGG
ubr-1	UBR-1 LD1	ATGCATACTATGTGAACACG **TGG** (reverse strand)	GATTATAGAATAATGAACCTTCGATAACACTCATATGCCATACTAGCTGAAGCCG**T**T**G**AAACATCAACTCCATATTGAAGTGTAGCTGGTGCTG (reverse complement)
ubr-1	UBR-1 LD2	GACGTGTATTCTGTGTCAAG **AGG**	GGCACGCGGAGCTCGTGAAACCCCGAACATTGACGTCAATTCTGTGTCAAG**A**A**G**ATGAAATAATTGCACCGCAACAGGGAAAACCAATGG
got-2.2	GOT-2.2 3-frame stop	CCGCCGTGCGAGGAAAGTCG **TGG**	CCAAGAAGCTTTTCTCTAACCGCCGTGCGAGGAAAGGACTACAAAGACCATGACGGTGATTATAAAGATCATGACATCGATTACAAGGATGACGATGACAAGTGACTATGAAGTGATAAGCTAGCTCG**TGG**TGGTCGCATGTTGAGATGGGACCACCAGATGCGAT
gln-3	GLN-3 3-frame stop	TTCCGGCGTAGAGGCATGCG **CGG** (reverse strand)	TGGTTCCCGAGATCTGGATTCCGGCGTAGAGGCATGCTAGCTTATCACTTCATAGTCACTTGTCATCGTCATCCTTGTAATCGATGTCATGATCTTTATAATCACCGTCATGGTCTTTGTAGTCTGCG**CGG**TAGTGAGCCTCGACGATGTCGCGACCGTAGACCTT (reverse complement)

Legend: underline (crRNA targeting sequence), **bold** (Pam sequence), Green: insertions, Red: Substitutions, Blue: silent mutations in repair to prevent Cas9 re-cutting.

bioRxiv preprint doi: https://doi.org/10.1101/2025.07.28.666006; this version posted July 30, 2025. The copyright holder for this preprint (which was not certified by peer review) is the author/funder, who has granted bioRxiv a license to display the preprint in perpetuity. It is made available under aCC-BY 4.0 International license.

Supplementary Table 7 – injection conditions

Figure	Transgene/Gene edit	Injected Strain	Injection Mix
Figs 2, 3, 6, Supp Figs 7, 8, 11	GFP::UBR-1	N2	5.0 uL Cas9 (10 µg/µl)
			5.0 uL IDT tracrRNA (4 µg/µl) in Duplex Buffer
			0.4 ul dpy-10 crRNA (from IDT) (4 µg/µl)
			2.0 ul GFP::UBR-1 crRNA (4 µg/µl)
			5 µl GFP::UBR-1 repair oligo (4 µg)
			0.55 µl dpy-10 repair ssODN, 500 ng/µl
			2.05 µl H_2O
Supp Figs 7, 8	UBR-1::GFP	N2	5.0 uL Cas9 (10 µg/µl)
			5.0 uL IDT tracrRNA (4 µg/µl) in Duplex Buffer
			0.4 ul dpy-10 crRNA (from IDT) (4 µg/µl)
			2.0 ul UBR-1::UBR-1 crRNA (4 µg/µl)
			5 µl UBR-1::GFP repair oligo (4 µg)
			0.55 µl dpy-10 repair ssODN, 500 ng/µl
			2.05 µl H_2O
Fig 1, Supp Fig 8	UBR-1 LD1	N2/ *ubr-1*(*bgg110*[GFP::UBR-1 CRISPR]) I	5.0 uL Cas9 (10 µg/µl)
			5.0 uL IDT tracrRNA (4 µg/µl) in Duplex Buffer
			0.56 ul dpy-10 crRNA (from IDT) (4 µg/µl)
			2.0 ul LD1 crRNA (4 µg/µl)
			2.2 ul LD1 repair oligo (1 µg/µl)
			0.55 µl dpy-10 repair ssODN, 500 ng/µl
			4.69 µl H_2O
Fig 1	UBR-1 LD2	N2	5.0 uL Cas9 (10 µg/µl)
			5.0 uL IDT tracrRNA (4 µg/µl) in Duplex Buffer
			0.56 ul dpy-10 crRNA (from IDT) (4 µg/µl)
			2.0 ul LD2 crRNA (4 µg/µl)
			2.2 ul LD2 repair oligo (1 µg/µl)
			0.55 µl dpy-10 repair ssODN, 500 ng/µl
			4.69 µl H_2O
Fig 3, Supp Fig 7	GFP::SL2::UBR-1 CRISPR	*ubr-1*(*bgg110*[GFP::UBR-1 CRISPR]) I	5.0 uL Cas9 (10 µg/µl)
			5.0 uL IDT tracrRNA (4 µg/µl) in Duplex Buffer
			0.56 ul dpy-10 crRNA (from IDT) (4 µg/µl)
			2.0 ul LD2 crRNA (4 µg/µl)
			2.2 ul LD2 repair oligo (1 µg/µl)
			0.55 µl dpy-10 repair ssODN, 500 ng/µl
			4.69 µl H_2O
Figs 4, 5, 6 Supp Fig 10	GOT-2.2 3-frame stop CRISPR	N2/*ubr-1*(*tm5996*) I	5.0 uL Cas9 (10 µg/µl)
			5.0 uL IDT tracrRNA (4 µg/µl) in Duplex Buffer
			0.56 ul dpy-10 crRNA (from IDT) (4 µg/µl)
			2.0 ul LD2 crRNA (4 µg/µl)
			2.2 ul LD2 repair oligo (1 µg/µl)
			0.55 µl dpy-10 repair ssODN, 500 ng/µl
			4.69 µl H_2O
Figs 4, 5, 6 Supp Fig 10	GLN-3 3-frame stop CRISPR	N2/*ubr-1*(*tm5996*) I	5.0 uL Cas9 (10 µg/µl)
			5.0 uL IDT tracrRNA (4 µg/µl) in Duplex Buffer
			0.56 ul dpy-10 crRNA (from IDT) (4 µg/µl)

bioRxiv preprint doi: https://doi.org/10.1101/2025.07.28.666006; this version posted July 30, 2025. The copyright holder for this preprint (which was not certified by peer review) is the author/funder, who has granted bioRxiv a license to display the preprint in perpetuity. It is made available under aCC-BY 4.0 International license.

			2.0 ul LD2 crRNA (4 µg/µl)
			2.2 ul LD2 repair oligo (1 µg/µl)
			0.55 µl dpy-10 repair ssODN, 500 ng/µl
			4.69 µl H_2O
Fig 1	P_{ubr-1}::UBR-1::ubr-1 3'UTR, unc-119(+) II	ttTi5605 II; unc-119(ed3) III; oxEx1578	pBG-GY1139 (MosSci (unc-119) ttTi5605 Pubr-1(3 kb)::ubr-1 genomic::ubr-1 3'UTR) 10 ng/µl
			pCFJ601 (Peft-3::transposase) 50 ng/µl
			pMA122 (Phsp::peel-1) 10 ngµl
			pGH8 (Prab-3::mcherry) 10 ng/µl
			pCFJ90 (Pmyo-2::mcherry) 2.5 ng/µl
			pCFJ104 (Pmyo-3::mcherry) 5 ng/µl
Fig 2	P_{glr-1}::UBR-1::let-858 3'UTR, unc-119(+) II	ttTi5605 II; unc-119(ed3) III; oxEx1578	pBG-GY1143 (MosSci (unc-119) ttTi5605 Pubr-1(3 kb)::ubr-1 genomic::ubr-1 3'UTR) 10 ng/µl
			pCFJ601 (Peft-3::transposase) 50 ng/µl
			pMA122 (Phsp::peel-1) 10 ngµl
			pGH8 (Prab-3::mcherry) 10 ng/µl
			pCFJ90 (Pmyo-2::mcherry) 2.5 ng/µl
			pCFJ104 (Pmyo-3::mcherry) 5 ng/µl

Supplementary Movie Legends

Supplementary Movie 1: High-intensity locomotor behavior is reduced in *ubr-1* mutants – Example 1. 20-second movie showing swimming for wild type (left) and *ubr-1(tm5996)* mutants (right). Note reduced swimming speed and abnormal tail movement in *ubr-1* mutants. Black box highlights animal where still frames are shown from this movie (00:04-00:05 sec) in **Figure 1B**.

Supplementary Movie 2: High-intensity locomotor behavior is reduced in *ubr-1* mutants – Example 2. Second 20-second movie showing swimming for wild type (left) and *ubr-1(tm5996)* mutants (right).

Supplementary Movie 3: Example of low-intensity locomotion displayed by wild-type animals on plates. 2-minute movie showing wild-type animals crawling on solid media.

bioRxiv preprint doi: https://doi.org/10.1101/2025.07.28.666006; this version posted July 30, 2025. The copyright holder for this preprint (which was not certified by peer review) is the author/funder, who has granted bioRxiv a license to display the preprint in perpetuity. It is made available under aCC-BY 4.0 International license.

Supplementary Movie 4: Example of normal forward low-intensity locomotion of *ubr-1* mutants on plates. 2-minute movie showing *ubr-1(tm5996)* mutants crawling on solid media. Note forward locomotor defects are not apparent.

Supplementary Movie 5: Example of 3D reconstruction of super-resolution imaging for CRISPR engineered GFP::UBR in the head of *C. elegans*.

Supplementary Movie 6: Example of 3D reconstruction of super-resolution imaging for the head of wt *C. elegans* control.
